# Novel topological descriptors for analyzing biological networks

**DOI:** 10.1186/1472-6807-10-18

**Published:** 2010-06-17

**Authors:** Matthias M Dehmer, Nicola N Barbarini, Kurt K Varmuza, Armin A Graber

**Affiliations:** 1 Institute for Bioinformatics and Translational Research, UMIT, Eduard Wallnoefer Zentrum 1, A-6060, Hall in Tyrol, Austria; 2 Department of Computer Science and Systems, University of Pavia, Via Ferrata 1, 27100, Pavia, Italy; 3 Institute of Chemical Engineering, Laboratory for Chemometrics, Vienna University of Technology, Getreidemarkt 9/166, A-1060 Vienna, Austria

## Abstract

**Background:**

Topological descriptors, other graph measures, and in a broader sense, graph-theoretical methods, have been proven as powerful tools to perform biological network analysis. However, the majority of the developed descriptors and graph-theoretical methods does not have the ability to take vertex- and edge-labels into account, e.g., atom- and bond-types when considering molecular graphs. Indeed, this feature is important to characterize biological networks more meaningfully instead of only considering pure topological information.

**Results:**

In this paper, we put the emphasis on analyzing a special type of biological networks, namely bio-chemical structures. First, we derive entropic measures to calculate the information content of vertex- and edge-labeled graphs and investigate some useful properties thereof. Second, we apply the mentioned measures combined with other well-known descriptors to supervised machine learning methods for predicting Ames mutagenicity. Moreover, we investigate the influence of our topological descriptors - measures for only unlabeled vs. measures for labeled graphs - on the prediction performance of the underlying graph classification problem.

**Conclusions:**

Our study demonstrates that the application of entropic measures to molecules representing graphs is useful to characterize such structures meaningfully. For instance, we have found that if one extends the measures for determining the structural information content of unlabeled graphs to labeled graphs, the uniqueness of the resulting indices is higher. Because measures to structurally characterize labeled graphs are clearly underrepresented so far, the further development of such methods might be valuable and fruitful for solving problems within biological network analysis.

## Background

Major reasons for the emergence of biological network analysis [1-4] are the extensive use of computer systems during the last decade and the availability of highly demanding and complex biological data sets. For instance, important types of such biological networks are protein-protein interaction networks [5-7], transcriptional regulatory networks [8,9], and metabolic networks [7,10,11]. Note that vertices in such biological networks can represent, e.g., proteins, transcription factors or metabolites which are connected by edges representing interactions, concentrations or reactions, respectively [3,12]. Thus, vertex-and edge-labeled graphs is an important graph class [13,14] and useful for modeling biological networks [3]. To name only some well-known examples or methods which have often been applied within biological network analysis, we briefly mention graph classes like scale-free and small-world networks [15,16], network centralities [12,17], module and motif detection [18-20], and complexity measures for exploring biological networks structurally [21,22].

Taking into account that a large number of graph-theoretical methods have been developed so far, approaches to process and meaningfully analyze labeled graphs are clearly underrepresented in the scientific literature. In particular, this holds for chemical graph analysis where various graph-theoretical methods and topological indices have been intensely used, see, e.g., [23-34]. Yet, we state a few examples where such graphs appear in the context of biological network analysis: Structure descriptors to determine the complexity of pathways representing labeled graphs have been used to examine the relationship between metabolic and phylogenetic information, see [22]. Another challenging task relates to determine the similarity between graphs or subgraphs [35-38]. For instance, YANG et al. [38] recently developed path-and graph matching methods involving vertex-and edge-labeled graphs which turned out to be useful for biological network comparison [38]. Finally, to utilize graph-theoretical concepts for investigating graphs and labeled graphs within molecular biology, HUBER et al. [39] reviewed several existing software packages and outlined concrete applications [39].

In this paper, we restrict our analysis to a set of bio-chemical graphs which have already been used for predicting Ames mutagenicity, see [40]. To perform this study, we develop and investigate entropic descriptors for vertex- and edge-labeled graphs. Before sketching the main contributions of our paper, we state some facts about topological descriptors which have been used in mathematical chemistry, drug design, and QSPR/QSAR.

As already mentioned, topological indices have been proven to be powerful tools in drug design, chemometrics, bioinformatics, and mathematical and medicinal chemistry [23,24,26,28,29,34,41-43]. Certainly, one reason for their success can be understood by the fact that there is a strong need to apply empirical models to solve QSPR (Quantitative structure-property relationship)/QSAR (Quantitative structure-activity relationship) problems [24,28,29,44] and related tasks in the just mentioned areas. In this paper, we put the emphasis on developing novel molecular descriptors for tackling a problem in QSAR: We will use structural property descriptors of molecules based on SHANNON's entropy for predicting Ames mutagenicity, see [40,45-47]. Generally, we note that the problem of detecting mutagenicity in vitro is based on the bacterial reverse mutation assay (Ames test) and often serves as a crucial tool in drug design and discovery [40,45-47].

Further, topological descriptors have often been combined with other techniques from statistical data analysis, e.g., clustering methods [26,48] to infer correlations between the used indices. Besides using topological descriptors for characterizing chemical graphs [27,32,49], they have also been applied to quantify the structural similarity of chemicals representing networks [50,51]. Among the large number of existing topological indices, an important class of such measures relies on SHANNON's entropy to characterize graphs by determining their structural information content [27,52-54]. Until now, especially these measures have been intensely applied within biology, ecology, and mathematical chemistry [27,52,54-60], in particular, to measure the complexity of biological and chemical systems [27,52,61]. Recently, we already developed a novel procedure to infer such information-theoretic measures for graphs that results in so-called partition-independent measures [57,62]. More precisely, we mean that we do not induce partitions using the procedure manifested by Equation (2), (3) in [57]. In this work, partitions using graph invariants and equivalence criteria have been explicitly induced, see, e.g., [27,52,53]. Note that we already placed a comment on this problem in the first paragraph of the section 'Partition-Independent Information Measures for Graphs'. In contrast to partition-independent measures, classical partition-based information measures often rely on the problem to group elements manifested by an arbitrary graph invariant according to an equivalence criterion [27,53,54,63].

**The contribution of our paper is twofold: **First, we develop some novel information-theoretic descriptors having the ability to incorporate vertex- and edge-labels when measuring the information content of a chemical structure. Because we already mentioned that there is a lack of graph measures which can process vertex-and edge-labeled graphs meaningfully, such descriptors need to be further developed. In terms of analyzing chemical structures, that means they can only be adequately represented by graphs if different types of atoms (vertices) and different types of bonds (edges) are considered. Hence, there is a strong need to exploring such labeled networks. Besides developing the novel information-theoretic measures for vertex- and edge-labeled graphs, we will investigate some of their properties thereof (see section 'Properties of the Novel Information-Theoretic Descriptors') [40,47]. Second, the paper also deals with evaluating the ability of the mentioned descriptors to predict Ames mutagenicity when applying well-known machine learning methods like random forests [64,65] (RF) and support vector machines [64,66] (SVM). Starting from chemical structures represented as vectors composed of topological descriptors, we will analyze the prediction performance by focussing on the underlying supervised graph classification problem. We want to emphasize that beside our novel descriptors, we also combine them with other well known information-theoretic and non-information-theoretic measures which turned out to be useful in QSPR/QSAR, see, e.g., [29]. Further, we examine the influence on the prediction performance when taking semantical (labels) and structural information of the graphs into account. Finally, we want to point out that considerable related work has been done so far that deals with investigating multifaceted problems when applying molecular descriptors to machine learning algorithms [67-69]. For example, DESHPANDE et al. [67] developed an approach to find discriminating substructures of chemical graphs. Then, by using a vector representation model for these graphs, they applied several machine learning methods to chemical databases for classifying these structures meaningfully. Another interesting study was done by XUE et al. [68] that deals with applying a variety of molecular descriptors to characterize structural and physicochemical properties of molecules [68]. Particularly, they used a feature selection method for automatically selecting molecular descriptors for SVM-prediction of P-glycoprotein substrates and others. As an important result, XUE et al. [68] determined the reduction of noise and its influence on the prediction accuracy of a statistical learning system [70]. The last contribution we want to sketch in brief is due to MAHÉ et al. [69]. In this work, a graph kernel approach [64,69] was validated for structure-activity-relationship analysis where special kernels based on random walks were used and optimized. Note that more related work can be found in [40,71-74].

## Methods

### Graph-Theoretical Preliminaries

To present the novel information-theoretic measures for labeled (weighted) graphs, we express some graph-theoretical preliminaries [14,57,75-77].

**Definition 1 ***is a finite, undirected graph. In this paper, we always assume that the considered graphs are connected and do not have loops*.

**Definition 2 ***Let G be a finite and undirected graph*. *δ*(*v*) *is called the degree of a vertex v ∈ V and equals the number of edges e ∈ E which are incident with v*.

**Definition 30 ***d*(*u*, *v*) *stands for the distance between u ∈ V and v ∈ V expressed as the minimum length of a path between u,v. Further, the quantity σ*(*v*) = max_*u*∈*V *_*d*(*u*, *v*) *is called the eccentricity of v ∈ V*. *ρ*(*G*) = max_*v*∈*V *_*σ*(*v*) *is called the diameter of G*.

**Definition 4 ***We call*(1)

*the j-sphere of a vertex v*_*i *_*regarding G*.

**Definition 5 ***Let*(2)

and(3)

*be unique (finite) vertex and edge alphabets, respectively*. *and **are the corresponding edge and vertex labeling functions. G *:= (*V*,*E*,*l*_*V*_,*l*_*E*_) *is called a finite, labeled graph*.

**Definition 6 ***Let*(4)

*Clearly*, *denotes the cardinality of the set of vertices whose distances, starting from v_*i*_, are equal to j and possess the vertex label *.

To finalize this section, we repeat the definition [76] of a so-called local information graph of an undirected graph *G*. In the following, we will use this definition to derive an advanced information functional for incorporating edge- and vertex-labels when measuring the structural information content of a labeled network.

**Definition 7 ***Let G *= (*V*, *E*) *be an undirected graph. For a vertex v*_*i *_∈ *V, we calculate **and the induced shortest paths*,(5)(6)(7)

*k*_*j *_*stands for the number of shortest paths of length j. Their edge sets are defined by*(8)(9)(10)

Further, let(11)

and(12)

*The local information graph *ℒ_*G*_(*v*_*i*_, *j*) *of G regarding v*_*i *_*is finally defined by*(13)

**Figure 1:**
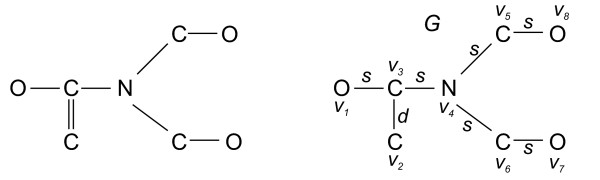
**A chemical structure and its corresponding labeled graph version**.

**Figure 2:**
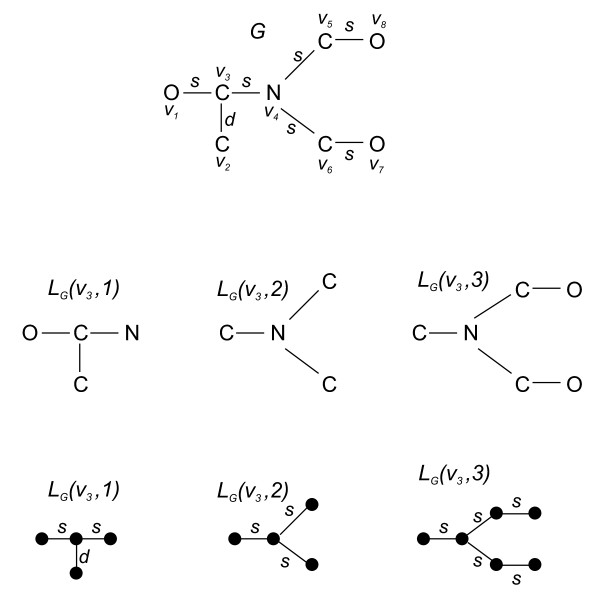
**A labeled chemical graph and its local information graphs regarding the vertex *v*_3_**. To understand the procedure for computing the structural information content of such a graph, the determined local information graphs are depicted as vertex-labeled (to incorporate vertex labels) as well as edge-labeled (to incorporate edge labels) graphs.

Fig. 1 shows a chemical structure as a labeled graph whereas Fig. 2 illustrates Definition (7).

### Partition-Independent Information Measures for Graphs

As already outlined, the majority of classical information measures for graphs are based on determining partitions by using an arbitrary graph invariant and an equivalence criterion, see, e.g., [27,48,53,54]. However, DEHMER et al. [57,62] recently proposed another method for quantifying the structural information content of a graph. The key principle of this approach is to assign a probability value to every vertex in a graph using different information functionals [57,62]. This results in partition-independent information measures to determine the entropy of the underlying graph topology. We already explained why we call our measures partition-independent (see also the section 'Background'). In a narrow sense, one might argue that to calculate the information functionals  and *f *^*E *^(see next section), we also deal with certain graph partitions for quantifying the information content of a vertex- and edge-labeled graph because we have to compute all local information graphs (local subgraphs). But nonetheless, the construction of our information measures basically differs from the ones mentioned in [57] (see Equation (2), (3)). In fact, we end up with probability values for every vertex of a given graph. Now, in order to start developing the new measures, we briefly recall the most important definitions. A recent review on information-theoretic descriptors to quantify structural information of unlabeled graphs can be found in [57].

**Definition 8 ***Let G *= (*V*, *E*) *be an arbitrary finite graph. The vertex probabilities for each v *_*i *_∈ *V are defined by the quantities*(14)

*f represents an arbitrary information functional*.

**Definition 9 ***Let G *= (*V*, *E*) *be an arbitrary finite graph. Then, the entropy of G is defined by*(15)

Now, we repeat the definition of an information functional for quantifying the structural complexity of unlabeled and unweighted chemical graphs [57]. Generally, this relates to measure the structural information content of a graph that is interpreted as the entropy of the underlying graph topology.

**Definition 10 ***Let G *= (*V*, *E*) *be an undirected finite graph. For a vertex v *_*i *_∈ *V, the information functional f *^*V *^*is defined as*(16)

**Remark 1 ***We want to point out that further information functionals have been developed so far *[76]. *The appropriateness of such a functional that captures structural information of a graph strongly depends on the graph class and on the specific problem under consideration*.

Another measure to determine the structural information content is the following one. Until now, it has been used [57] to perform a statistical analysis when determining structural complexity of real chemical structures and investigating correlations with other molecular descriptors [57]. Mathematical properties thereof were also described in [57].

**Definition 11 ***Let G *= (*V*, *E*) *be an undirected finite graph. We define the family of information measures*(17)

where(18)

λ > 0 *is a scaling constant*.

### Novel Information-Theoretic Descriptors for Labeled Graphs

In this section, we present novel information measures to quantify structural information of labeled (weighted) chemical structures by adapting the just shown approach. Because the majority of the developed topological indices is only defined for the underlying skeleton of a chemical structure, the further development of descriptors for processing chemical graphs containing heteroatoms and multiple bonds is generally of great importance. Before we start expressing the new definitions, we first point out some related work in this area.

Note that earlier contributions to infer measures for labeled graphs are often based on special distance matrices and polynomial methods [78-80]. Another attempt in this direction was done by IVANCIUC et al. [81] where this approach is based on defining weighted matrices incorporating special weighting schemes [81]. For example, a definition of a connectivity, adjacency, distance, and reciprocal distance matrix by applying several weighting schemes incorporating chemical information like the atomic bond number, electronegativity, and the covalent radius have been investigated [81]. Then, such matrices have been used to define molecular descriptors for quantifying information of weighted chemical graphs, e.g., organic compounds. To name some concrete examples, we first mention the WIENER index [82] for vertex-and edge-labeled graphs when applying the known formula for calculating this index with a special weighting scheme as mentioned above [81]. Further, starting from the mentioned weighted matrices, the well-known information indices *U*, *V*, *X*, *Y *[83] have been extended to determine the structural information content of labeled (weighted) graphs [84]. As a result, IVANCIUC et al. [81,84] obtained information-theoretic topological descriptors for vertex- and edge-labeled graphs where the underlying (weighted) matrix may contain negative elements and those between zero and one.

We now start by stating the novel partition-independent information-based descriptors to determine the information content of vertex- and edge-labeled graphs. The first definition represents an information functional to account for vertex labels of a chemical structure. For this, we adapt the idea [57,62] of determining the topological neighborhoods (using *j*-spheres) for all involved atoms (vertices) of the molecule. By now considering labeled graphs, our first attempt results in an information functional with the property that every vertex in each *j*-sphere possessing a certain vertex label (atom type) will be weighted differently.

**Definition 12 ***Let G *= (*V*, *E*, *l*_*V*_) *be an undirected finite vertex-labeled graph*, . *We define*(19)

**Example 2 ***To demonstrate the calculation of ** exemplarily, we consider Fig*. 1*and set *. *O, C and N denote the atom types of the molecule. The edge type s represents a single bond whereas d represents a double bond within the chemical structure. For example, if we now calculate ** for G shown in Fig*. 1, *we yield*(20)(21)(22)(23)(24)(25)(26)(27)

Because it is not always clear how to choose the involved parameter in practice, we further derive an information functional to overcome this problem.

**Definition 13 ***Let G *= (*V*, *E*, *l*_*V*_) *be an undirected finite vertex-labeled graph*, . *If we determine all local information graphs *ℒ_*G*_(*v*_*i*_, *j*) *of G for the vertices v*_*i *_∈ *V, we then define the quantities*(28)

*This quantity denotes the number of vertices of *ℒ_*G*_(*v*_*i*_, *j*) *possessing vertex label *.

**Definition 14 ***Let G *= (*V*, *E*, *l*_*V*_) *be an undirected finite vertex-labeled graph*, . *We define the information functional*(29)

*where *.

**Remark 3 ***We note that*(30)(31)

The expression(32)

*quantifies the number of occurrences of vertex label **in*(33)

**Example 4 ***Fig*. 2*shows the calculated local information graphs of G regarding v*_3_. *For example, this leads to*(34)

*By determining all local information graphs for the remaining vertices of G, the just shown calculation can be performed analogously*.

Next, we are able to derive an information functional that takes the edge labels of a graph *G *into account. The main idea is to use weighted paths which can be directly determined by calculating the local information graphs.

**Definition 15 ***Let G *= (*V*, *E*, *l*_*E*_) *be an undirected finite edge-labeled graph*, *, and assume that there exists a correspondence between the edge labels and numerical values. We define*(35)

where(36)

and(37)

Now, we present an example how to apply this definition to the local information graphs shown in Fig. 2.

**Example 5 ***We exemplarily apply the information functional f *^*E *^*to G and v*_3 _*as the starting vertex and recall that s *= 1, *d *= 2. *The edge labeled local information graphs for this vertex are depicted in Fig*. 2. *We yield*,(38)

and(39)(40)(41)

*Thus*,(42)

In order to incorporate both edge and vertex labels when determining the topological entropy of a labeled graph, we also derive

Definition 16(43)(44)

Finally, we obtain the following entropy measures for measuring the structural information content of labeled graphs.

**Definition 17 ***Let G *= (*V*, *E*, *l*_*V*_, *l*_*E*_) *be an undirected finite labeled graph*, . *We now straightforwardly define the information-theoretic descriptors (graph entropy measures) as follows:*(45)(46)(47)(48)(49)

**Remark 6 ***We emphasize that according to the above stated definition and the definitions of the underlying information functionals, the resulting information measures are obviously parametric. This property generalizes classical information measures which have often been used in mathematical chemistry, see, e.g.*, [27,29,53,83]. *As already pointed out in *[57], *such measures establish a link to machine learning because the parameters could be learned using appropriate datasets. However, we won't study this problem in the present paper*.

## Results and Discussion

This section aims to evaluate the just presented (see previous section) information measures for labeled graphs numerically. Also, we will calculate some known information indices to tackle the second part of our study when applying these measures to machine learning algorithms. Our study will be twofold: First, we examine some properties of the measures for labeled graphs when applying them to a large set of real chemical structures. Second, we analyze a QSAR problem by applying supervised machine learning methods [64,85] using our novel molecular descriptors.

### Data

We created the database AG 3982 from the benchmark database called Ames mutagenicity [40,47] originally used for the evaluation and prediction of the mutagenicity of chemical compounds [40]. The Ames database was created from six different public sources [40,47] and each chemical structure possesses a class label (0 and 1) that results from the Ames test indicating the genetoxicity of a substance. By starting from the original database Ames mutagenicity [40,47] containing 6512 chemical compounds, we created AG 3982 by filtering out isomorphic graphs based on the software SubMat [86]. Finally, this procedure resulted in 3982 structurally different skeletons, that is, all atoms and all bonds are considered as equal. Among these 3982 graphs, 1794 possess class label 0 and 2188 possess 1. It holds 2 ≤ |*V*| ≤ 109; 1 ≤ *ρ*(*G*) ≤ 47 ∀ *G *∈ AG 3982. To evaluate the novel descriptors for labeled graphs, we then considered these structures as vertex- and edge-labeled graphs. Evidently, for calculating the descriptors of the unlabeled graph versions (skeletons), the corresponding descriptors were used which take only topological information into account.

### Technical Processing of the Structures and Software

To generate and process the underlying graph structures, we used the known Molfile format [71]. The graphs from AG 3982 were originally available in Smiles format that we converted to Molfile format (SDF) using a Python procedure. The implementation of all topological descriptors based on the Molfile format was performed by Python using freely available libraries like Networkx, Openbabel and Pybel packages [87]. To perform the graph classification using random forests (RF) [64,66] and support vector machines (SVM) [64,66], we used the implementations provided by the Python package Orange [88]. The feature selection was done by Weka [89].

### Properties of the Novel Information-Theoretic Descriptors

Before starting to evaluate our novel molecular descriptors, we define some concrete information measures by choosing special weighting schemes for the coefficients.

**Definition 18 ***We define a special weighting scheme for the coefficients **to determine **as follows: Starting from*(50)

*where m*_*a *_*denotes the atomic mass of the atom a (in the i-th sphere), we also define*(51)

*The scheme starts with the lightest element Hydrogen (H) and ends with the heaviest one, namely Uranium (U). If the underlying c*_*i *_*will be chosen by*(52)

*and by using Definition (11) and Definition (17), the concrete information-theoretic descriptors are called **and *. *If the underlying c*_*i *_*will be chosen by*(53)

*the measures **and **follow correspondingly. Further, if the underlying c*_*i *_*will be chosen linearly or exponentially decreasing (in both functionals **and f *^*E*^*); see also that the measures **and **follow correspondingly (Equation (50), (35), (52), (53))*.

**Definition 19 ***Let G *= (*V*, *E*, *l*_*V*_, *l*_*E*_) *be an undirected finite labeled graph*, . *If we choose the coefficients of information functional **(see Equation (29)) linearly or exponentially decreasing, we call the resulting information measures **and *.

**Figure 3:**
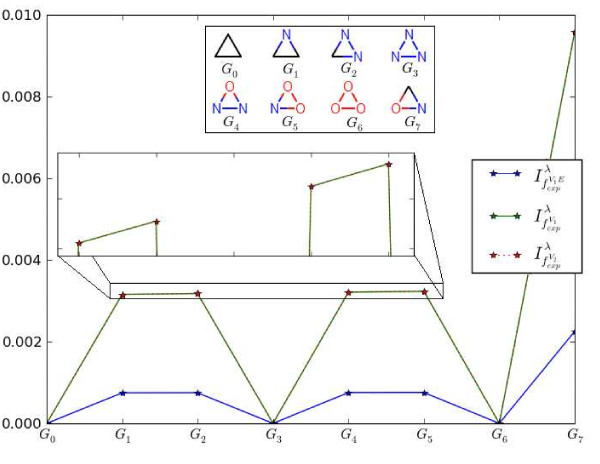
**Entropies vs. graph numbers for vertex-labeled graphs**.

**Figure 4:**
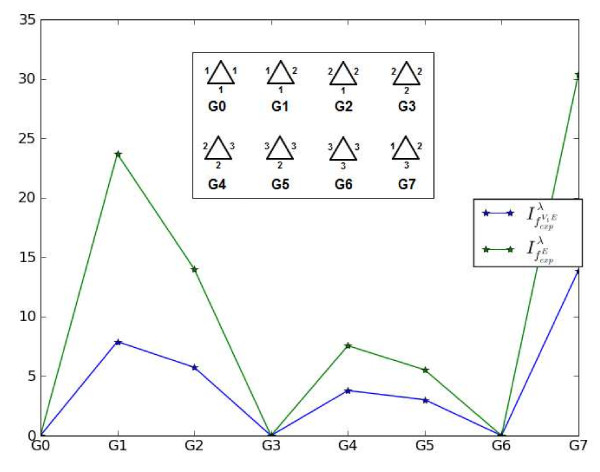
**Entropies vs. graph numbers for vertex- and edge-labeled graphs**.

Note, that we set *λ *= 1000 to perform the entire numerical calculations in this paper. In order to interpret some of these measures, we consider Fig. 3. As example graphs, we chose vertex-labeled cyclic graphs (all edge labels (weights) that correspond to bond types are equal to one). We note that independent from the chosen parameters, we have already shown [57] that for some vertex-transitive graphs like several *k*-regular graphs, the measure *I*_*f*_*v *always leads to maximum entropy. By definition, it then follows that . Taking this into account, it is evident that for *G*_0_, *G*_3 _and *G*_6_, all three measures vanish. Because the graphs *G*_1_, *G*_2 _and *G*_4_, *G*_5 _have different label configurations - based on the different weighting schemes - and, therefore, the line between these points is not exactly horizontal as shown by the zoomed region depicted in Fig. 3. Interestingly, the fact that the curves for  and  are equal is no coincidence and can be easily understood by observing that the underlying graphs only possess one sphere for every vertex. This implies that there is no difference when calculating the resulting the information measures. In summary, we see that the descriptors possess maximal values if all vertices have different atom types. Hence, we conclude that the more disordered the label configuration of the graph is, the lower is the value of *I*_*f*_*v *and the higher the value of . These observations are likewisely applicable to interpret Fig 4. This figure shows the structural information contents if we incorporate both different vertex- and edge labels. Similarly, the application of the selected indices to *G*_0_, *G*_3 _and *G*_6 _leads to descriptor values equal to zero. Again, we obtain maximal values for the calculated indices when applying them to *G*_7 _because the edge and vertex configurations are most disordered.

**Figure 5:**
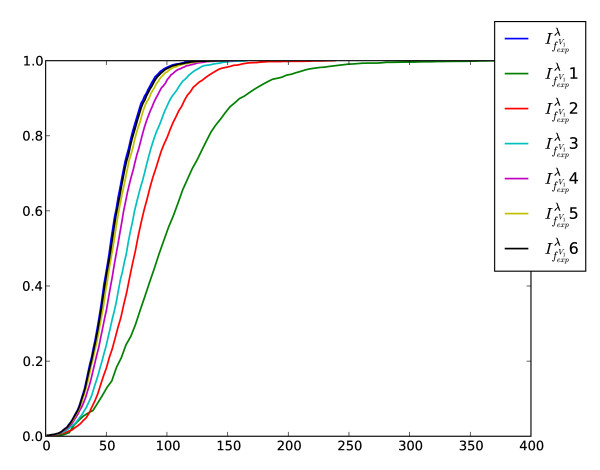
**Cumulative entropy distributions of the sphere-approximated measures using ****and exponentially decreasing coefficients**. The graphs of AG 3982 were treated as only vertex-labeled graphs. The *x*-axis is formed by calculating the values of the descriptors where the *y*-axis denotes the percentage rate of all graphs.

**Figure 6:**
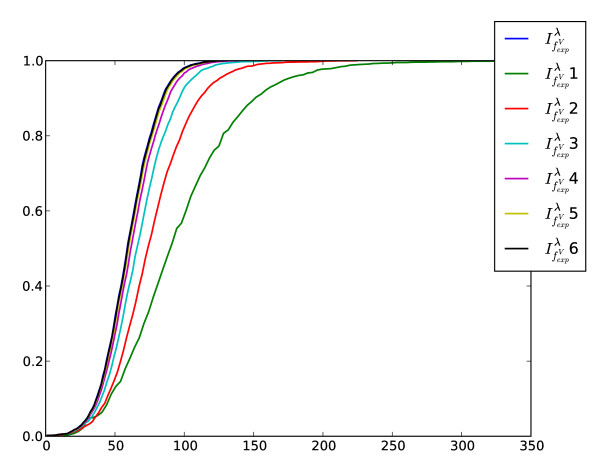
**Cumulative entropy distributions of the sphere-approximated measures using *f*^*V *^and exponentially decreasing coefficients**. The graphs of AG 3982 were treated as unlabeled graphs. As in the previous figure, the *x*-axis is formed by calculating the values of the descriptors where the *y*-axis denotes the percentage rate of all graphs.

Another problem we want to investigate relates to determine the information loss when computing the structural information content by truncating the cardinalities of the *j*-spheres. To determine the corresponding descriptor values, we first considered the graphs of AG 3982 as only vertex-labeled graphs (see Fig. 5). The notation  1 means we set  for *k *> 1;  2 implies that we set  for *k *> 2 etc. Thus, the measure *i *can be interpreted as an approximation that only takes the first *i*-th sphere cardinalities (for all atoms of the molecule) into account. If we use the information functional  to compute the information content of the vertex-labeled graphs, Fig. 5 shows that by incorporating the first five *j*-sphere cardinalities (for all atoms of the molecule), the resulting measure captures nearly the same structural information than . This can be understood by observing that the corresponding cumulative entropy distributions are almost equal. Clearly,  takes all spheres of the graphs into account. Fig. 6 shows a similar result when using *f*^*V*^, that is, we only considered the skeleton versions. The plot shows that in this case,  4 approximates  quite well because their cumulative entropy distributions look again very similar. Finally, this study might be useful to save computational time when applying the measures to large networks. Further, it might give valuable insights when designing novel information-theoretic measures based on calculating spherical neighborhoods.

In order to evaluate the uniqueness (also called degeneracy [24,55,59]) of some information-theoretic indices, we applied them to AG 3982. Recently, DEHMER et al. [57] utilized the sensitivity index developed by KONSTANTINOVA et al. [59],(54)

to evaluate the discrimination power of an index *I*. In general,  is the cardinality of  and  denotes the set of graphs  which can not be distinguished by an index *I*. In Table 1, *I*_*orb *_denotes the well-known *topological information content *developed by RASHEVSKY[54] that is based on determining topologically equivalent vertices (which form the vertex orbits) to infer a probability value for each obtained partition [27,53]. *W *is the WIENER index [82] and [55,83](55)(56)(57)

where(58)(59)(60)

**Table 1:** Sensitivity for AG 3982

Topological Index *I *	*S*(*I*)
	0.995981

	0.996986

	1.0

	0.996986

	1.0

	0.995982

	0.995982

*I*_*orb *_	0.074334

*I*_*D *_	0.938724

	0.947513

*W *	0.037920

*I*_*W *_	0.990959

The table shows the sensitivity index *S*(*I*) of the main topological indexes for AG 3982.

Here, we assume that the distance of a value *i *in the distance matrix appears 2*k*_*i *_times [27]. *μ *denotes the cyclomatic number [83]. To evaluate the discrimination power of the novel descriptors for vertex- and edge-labeled graphs, we look at Table 1. When applying the partition-independent measures  only to skeletons of AG 3982, we see that the sensitivity values are very high, i.e., the corresponding measures possess a high uniqueness. Further, by incorporating edge- and vertex labels, the underlying measures are able to discriminate all graphs uniquely and, hence, . This corresponds to our anticipation that if we incorporate semantical information like edge- and vertex labels, this leads to an increase of the sensitivity measure expressing the uniqueness of the molecular descriptor. We remark that the partition-based measure *I*_*W *_also discriminates the graphs of AG 3982 quite well. In contrast, the discrimination power of *W *and *I*_*orb *_is comparably very low.

### Evaluation of the Descriptors Using Supervised Machine Learning Methods

In the following, we evaluate our novel and other descriptors by applying them to supervised machine learning methods [64,66]. First, our aim is to determine the classification performance of the underlying graph classification problem, i.e., to predict mutagenicity when applying topological descriptors for unlabeled and labeled graphs using SVM and random forests. Second, we examine the influence on the prediction performance when taking semantical (labels) and structural information of the graphs into account. As expressed in a previous section, AG 3982 can be divided into two classes because every graph possesses a unique label (zero or one). Thus, we here deal with a two-class classification problem. Note that by starting from the same underlying benchmark dataset Ames mutagenicity [40,47], a related study has already been recently performed [40]. However, HANSEN et al. [40] used the full database (Ames mutagenicity) containing 6512 compounds, molecular descriptors (Dragon [90]) based on the 3D structure, and supervised machine learning methods (Gaussian processes, RF, SVM, KKN) to predict mutagenicity. In fact, the main goal of this study was to evaluate the prediction performance based on different implementations of the mentioned machine learning algorithms.

Now, before discussing the classification results, we first state some definitions.

**Definition 20 ***Let I*_1_,..., *I*_*m *_*be topological indices. The superindex of these measures is defined as *[91](61)

**Definition 21 ***Let G *= (*V*, *E*, *l*_*V*_, *l*_*E*_) *be an undirected finite labeled graph*, . *Then, each graph will be represented by*(62)

To perform the graph classification, we chose *SI *such that it consists of the twelve indices from Table 1 together with . Thus, *m *= 16. The measure *I*_*U *_is defined as [83](63)

and by Equation (58). Further, we state the definitions [92] for(64)(65)

where(66)

and(67)(68)

Now, based on the *SI*-representation (see Equation (62)) of a chemical graph, we tackle the mentioned graph classification problem using RF and SVM. The main steps were as follows:

• We performed 10-fold crossvalidation for both classification methods.

• When doing cross validation, we did a parameter optimization on the corresponding training sets. By using different kernels like linear polynomials, polynomials of higher degree etc., we found that the RBF kernel give the best results.

• The random forest was composed by fifty different trees.

• We performed the classification both with all features (information measures) and with only seven features  determined by running a feature selection algorithm based on greedy stepwise regression [93].

The classification results are shown in Table 2 where we calculated the statistical quantities [64] Accuracy (Acc.), Sensitivity (Sens.), Specificity (Spec.), Precision (Prec.), and F-Measure to evaluate the performance of the classifiers. The F-Measure is generally defined by(69)

**Table 2:** The results of classification using RF and SVM.

Classifier	Attributes	Acc.	Sens.	Spec.	Prec.	F-Measure
Random Forest	16	67.2	69.1	65.0	69.1	69.1

Random Forest	Best 7	65.5	68.3	62.0	68.7	68.5

SVM	16	68.2	80.1	53.7	67.9	73.5

SVM	Best 7	65.2	78.7	48.7	65.2	71.3

The results of the graph classification using RF and SVM are presented in this table. In particular every tested classifier is applied by using both all the descriptors and only the best seven. The main statistical quantities are calculated for the evaluation: Accuracy (Acc.), Sensitivity (Sens.), Specificity (Spec.), Precision (Prec.), and F-Measure

Taking into account that we classified only with (i) sixteen and (ii) seven information measures, we consider the classification results as feasible. One clearly sees that for both classifiers, the Precision and Sensitivity values - which are important quantities to evaluate the performance of the classification - are relatively high. Precision is the probability that the cases classified as positives are correctly identified where Sensitivity is the probability of positive examples which were correctly identified as such. The F-Measure defined as the harmonic mean of Precision and Sensitivity represents a single measure to evaluate the performance of the classifiers. By definition, the F-Measure varies between zero and one whereas one would represent the perfect and zero the worst classification result. We clearly see that by using SVM's, we reached values of F-Measure of over seventy percent which are the highest among all calculated ones. In order to examine the influence of incorporating vertex- and edge-labeled graphs on the prediction performance, we first present the following procedure and, then, the obtained results, see Table 3:

**Table 3:** Comparison of the graph classification using unlabeled and labeled graphs

Classifier	Attributes	Acc.	Sens.	Spec.	Prec.	F-Measure
Random Forest	7U	63.2	65.2	60.9	67.0	66.1

*σ*		0.77	1.02	0.83	0.67	0.79

Random Forest	5U + 2L	64.0	66.5	60.9	67.5	67.0

*σ*		0.88	1.46	1.87	0.97	1.15

SVM	7U	63.0	83.3	38.2	62.2	71.2

*σ*		1.23	2.66	4.92	1.32	1.67

SVM	5U + 2L	65.0	79.3	47.7	64.9	71.4

*σ*		0.88	1.07	2.37	0.90	0.97

The table contains the results of the graph classification applying the information-theoretic descriptors for vertexand edge-labeled graphs. Here, *U *indicates the usage of a measure only defined for unlabeled graphs and *L *indicates the usage of a measure for vertex- and edge-labeled graphs, respectively. *σ *denotes the standart deviation of the corresponding means.

• Note that in our previously presented classification, we used eleven indices for unlabeled graphs and five for vertex- and edge-labeled graphs. From this feature set, we generated ten subsets composed of seven randomly selected measures for unlabeled graphs (among the eleven), and ten subsets composed of five randomly selected measures for unlabeled graphs and two measures for vertex- and edge-labeled graphs (among five available).

• Based on these sets, we again performed 10-fold cross validation with RF and SVM and averaged the classification results.

As a result, Table 3 reflects that if we apply the information-theoretic descriptors for vertex- and edge-labeled graphs, this leads to very similar results (e.g., by considering F-Measure) as in case of only measuring skeletal (structural) information. The calculated standard deviations support this hypothesis. Based on our intuition, we would normally expect that by additionally incorporating semantical information (labels), the graphs can be distinguished more meaningfully. Therefore, the results from Table 3 are astonishing because incorporating the information-theoretic descriptors for vertex- and edge-labeled graphs did not lead to a significant improvement of the prediction performance.

To finalize our numerical section, we also present results when choosing a different representation model of the graphs. In the following, we do not characterize a graph by its structural information content and by its superindex. In contrast, we now represent every graph by a vector that indicates if the given graphs contains certain substructures. To achieve this, we used a database [94] of 1365 substructures and the software SubMat [86] for determining the substructures which are contained in a graph in question. Then, every graph is characterized by a binary vector possessing 1365 entries that indicate the appearance or non-appearance of a substructure. For evaluating the quality of the used machine learning models (RF and SVM), we first performed a feature selection algorithm by again using greedy stepwise regression [93]. As a result, we ended up with twenty features to run the classification. Based on a 10-fold crossvalidation procedure, the classification results are depicted in Table 4.

**Table 4:** Classification using the substructure method

Classifier	Attributes	Acc.	Sens.	Spec.	Prec.	F-Measure
Random Forest	Best 20	64.2	63.3	65.3	69.0	66.0

SVM	Best 20	64.3	70.7	56.6	66.5	68.5

Here the results of the graph classification using RF and SVM are shown. To represent the underlying graphs, we chose the explained substructure method.

By looking at the performance evaluation in Table 4, we see again that the representation model based on the superindex led to prediction results which are similar to the ones by applying the model using the appearance or non-appearance of a substructure (see Table 2). From Table 2 and Table 4, we see that if we apply RF and SVM to perform the graph classification, it seems that the used information indices to create the underlying superindex captures structural information of the graphs (contained in AG 3982) similarly than the model that is based on the substructures. But to give a reason why most of the performance measures (mainly F-Measure) in Table 2 are slightly higher than in Table 4, it is plausible to conjecture that the used topological descriptors might measure more complex structural features like branching and other types of structural complexity than only counting the contained substructures.

## Conclusions

This paper dealt with investigating several aspects of information-theoretic measures for vertex- and edge-labeled chemical structures. We now summarize the main results of the paper as follows:

• We already mentioned that the majority of the topological indices which have been developed so far are only suitable to characterize unlabeled graphs. By adapting the approach of deriving partition-independent information measures, we developed families of information-theoretic descriptors to incorporate vertex- and edge labels when measuring the structural information content of graphs. First, we did this by calculating spherical neighborhoods and distinguishing atom types for every sphere. For the resulting measures, we presented a weighting scheme for the vertices which takes chemical information of the graphs into account. Second, to reduce the number of parameters, we developed a simplified version based on the so-called local information graphs. Generally, these graphs are induced by shortest paths and provide information about the local information spread in a network. We here assume that information spreads out via shortest paths in the network [95]. By using this principle, we defined an information functional (see Equation (29)) that relies on calculating the occurrences of existing and unique vertex labels within the local information graphs and on determining weighted paths. In this paper, we did not give a formal analysis of the computational complexity of the underlying algorithm to compute the corresponding information measures. However, we point out that it is easy to prove that their computation requires polynomial time.

• Using the benchmark database AG 3982, we evaluated the novel information-theoretic descriptors to see how they capture structural information of the chemical graphs. Based on some characteristic properties [57] of the measures, we found that the higher the value of the final measure is, the more disordered is the label configuration of a graph in question. Another aspect we have studied relates to determine their high uniqueness, that is, their ability to discriminate graphs as unique as possible. As a result, we derived that the measures for calculating the information content of vertex- and edge-labeled graphs possess a very high discrimination power. In particular, the computation of two of those led to sensitivity values equal to one, i.e., the measures distinguished all the graphs uniquely.

• Another aim was to predict Ames mutagenicity when using supervised machine learning methods (RF and SVM) and representing the graphs by a vector consisting of topological descriptors (superindex). First, we performed the graph classification based on 10-fold crossvalidation and evaluated the quality of the learned models. Taking into account that we only used (i) 16 and (ii) 7 information measures for classifying the graphs, we obtained feasible results (by using SVM, we reached F-Measures of over seventy percent). However, another goal was to examine the influence of incorporating vertex- and edge-labels when measuring the prediction performance of the underlying graph classification problem. Here, we obtained the result that the prediction performance (by calculating the statistical performance measures) was very similar to the one we obtained by only measuring skeletal (structural) information. From this, interesting future work arises as follows: Because of the obtained results, it would be important to explore the developed measures for determining the structural information content (structural complexity) of the underlying vertex- and edge-labeled graphs in depth. This aims to investigate the measures such that the prediction performance could be significantly improved when applying them to the machine learning methods we have used in this paper. Another reason for the results shown in Table 3 could be certain characteristics of the underlying graphs which need to be analyzed more deeply. As further future work, we will use different datasets to determine the prediction performance of the novel measures. Moreover, we want to perform similar analyses by applying our novel descriptors combined with a large number of other well-known molecular descriptors to the same benchmark database. But this goes beyond the scope of this paper.

• As already mentioned (see section 'Introduction'), labeled graphs play an important role when analyzing biological networks. But because the theory of labeled graphs is not well developed so far (compared to the contributions which have been done towards unlabeled graphs), see, e.g., [29], a thorough investigation of methods for analyzing these graphs is therefore crucial. On the other hand, to gain information about the basic biological understanding when investigating biological networks, the problem of exploring their topology is essential [5-7]. Hence, there is a strong need to further investigate methods to analyze labeled graphs for solving problems in bioinformatics and systems biology [22,38,39].

Inspired from this study, we think that especially the development of further measures for labeled graphs can be an interesting and valuable attempt not merely to analyze QSPR/QSAR problems. Besides applying these measures to machine learning methods, we believe that the measures itself might be valuable for those who will investigate biological networks, see, e.g., [22]. In fact, if we incorporate also semantical information of the graphs (instead of only considering structural information), this may lead to more meaningful results when developing methods for characterizing graphs or predictive models to tackle problems in bioinformatics, systems biology, and drug design.

• As a conclusive remark, we argue from a mathematical point of view that a further development of the theory of labeled graphs will surely help to develop more sophisticated methods for analyzing biological networks, see, e.g., [2,22,38,39]. The next important step is to prove mathematical properties of such measures and to investigate their relatedness. In addition, there is a need to examine correlations to other existing topological indices numerically.

## Authors' contributions

All authors contributed equally to all aspects of the article. All authors read and approved the final manuscript.
